# Combinatorial Techniques to Efficiently Investigate and Optimize Organic Thin Film Processing and Properties

**DOI:** 10.3390/molecules18044120

**Published:** 2013-04-08

**Authors:** Florian Wieberger, Tristan Kolb, Christian Neuber, Christopher K. Ober, Hans-Werner Schmidt

**Affiliations:** 1 Macromolecular Chemistry I and Bayreuth Institute of Macromolecular Research (BIMF), University of Bayreuth, D-95447 Bayreuth, Germany; 2 Materials Science & Engineering, Cornell University, Ithaca, NY 14853, USA

**Keywords:** organic thin film, material composition gradient, ternary combinatorial library, lithography, quartz crystal microbalance

## Abstract

In this article we present several developed and improved combinatorial techniques to optimize processing conditions and material properties of organic thin films. The combinatorial approach allows investigations of multi-variable dependencies and is the perfect tool to investigate organic thin films regarding their high performance purposes. In this context we develop and establish the reliable preparation of gradients of material composition, temperature, exposure, and immersion time. Furthermore we demonstrate the smart application of combinations of composition and processing gradients to create combinatorial libraries. First a binary combinatorial library is created by applying two gradients perpendicular to each other. A third gradient is carried out in very small areas and arranged matrix-like over the entire binary combinatorial library resulting in a ternary combinatorial library. Ternary combinatorial libraries allow identifying precise trends for the optimization of multi-variable dependent processes which is demonstrated on the lithographic patterning process. Here we verify conclusively the strong interaction and thus the interdependency of variables in the preparation and properties of complex organic thin film systems. The established gradient preparation techniques are not limited to lithographic patterning. It is possible to utilize and transfer the reported combinatorial techniques to other multi-variable dependent processes and to investigate and optimize thin film layers and devices for optical, electro-optical, and electronic applications.

## 1. Introduction

Combinatorial optimization methods are widely used, above all in pharmaceutical research, to screen new molecules for their potential application as drugs [1,2,3,4,5], but the combinatorial investigations approach has been aggressively adopted by the field of materials research in recent years, too [6,7,8]. Combinatorial materials science pursues the objective of preparing a family of related samples in a single experiment, to investigate interacting parameters and to efficiently and quickly optimize materials and processes. Due to the continuously arising challenges in materials development, the use of variable gradients and their combination to so called combinatorial libraries has been driven forward. Important research areas of established combinatorial approaches include sensing materials, catalysis, electronic and functional materials, and biomaterials [9]. In the last decade research has addressed the investigation of properties of organic thin films in relation to certain variable gradients [10]. In general the film thickness itself is an important variable for film application. Thus to enable a combinatorial investigation, films of continuous thickness gradients were prepared [11]. Amis *et al*. have demonstrated the dewetting behavior of a polystyrene film by preparing a film thickness gradient versus a continuous temperature gradient arranged in a 2-D combinatorial library [12]. His group also investigated the phase separation of a thin film prepared from a polymer blend gradient in combination with a temperature gradient [13]. The film preparation of this polymer blend gradient was realized utilizing a custom-built setup. In addition to solution cast composition and layer thickness gradients, solvent-free prepared gradients utilizing physical vapor deposition (PVD) are well-known. Here combinatorial optimizations of electro-optical devices were investigated in a 2-D combinatorial library in regard to composition and layer thicknesses [14]. Another important parameter, especially for adhesion investigations, is the chemical surface treatment of a film, thus gradients of surface characteristics were developed. Such a surface modification was obtained via electron beam treatment of a poly(2-vinylpyridine) coated surface. This electron beam exposure dose gradient applied over a length of 5 cm generates a hydrophilicity gradient on the surface [15]. Matsuda *et al*. have shown another method for the preparation of a surface hydrophilization gradient with a poly(vinyl carbonate) coated film [16]. This film hydrolyzes gradually via continuous immersion in an aqueous NaOH solution.

A special field of interest regarding organic thin films is lithography. In the lithographic patterning process the resist film has to pass through several steps, e.g., film preparation, annealing steps, exposure, development, and etching. This multi-step process, on the other hand, gives a variety of variables which interact strongly with each other, such as resist composition, film thickness, annealing temperature, exposure dose, or development time, to name but a few. Furthermore, the engineering and manufacturing of new patterning tools [17] and the introduction of new materials [18] leads to a perpetual optimization of the operating process [19]. Thus, combinatorial investigations became an interesting approach for lithographic issues in the last decade. For instance, different compositions of a molecular glass photoresist were investigated in combinatorial PVD-prepared libraries as a function of exposure dose [20]. Another important variable for thin polymer films, especially in the lithographic context, but also in general, is the annealing (bake) time and temperature. Therefore degradation of poly(*tert*-butoxycarbonyloxystyrene) has been investigated with temperature and also bake time gradients [21]. Furthermore the bake steps applied to chemically amplified resist systems in the lithographic patterning process have to be precisely identified for the post exposure and post apply bake steps. Hence, for the optimization of resist performance both bake steps were performed as temperature gradients in research laboratories and applied in a manner orthogonal to each other [22]. Recently we have identified a synergistic effect for post exposure bake and resist composition. Therefore the composition gradient was applied perpendicular to the temperature gradient of post exposure bake and combined with an exposure dose gradient as a ternary gradient [23,24,25]. These different gradient preparation techniques for organic thin film investigations demonstrate impressively the fast and effective variable investigation in one experiment. The presented article summarizes our improved and newly developed combinatorial techniques for organic thin film investigations, the preparation of binary and ternary combinatorial libraries, and their characterization.

## 2. Results and Discussion

### 2.1. Combinatorial Techniques

For a combinatorial investigation typically gradients of materials and/or process variables are applied. However for new combinatorial approaches the existing techniques have to be adapted, the setups modified or new techniques developed just to fit a particular set of requirements. In the following sections we show adapted and newly developed combinatorial techniques and the corresponding setups focused on combinatorial approaches for solution-processed organic thin films.

#### 2.1.1. Internal Material Composition Gradient

**Figure 1:**
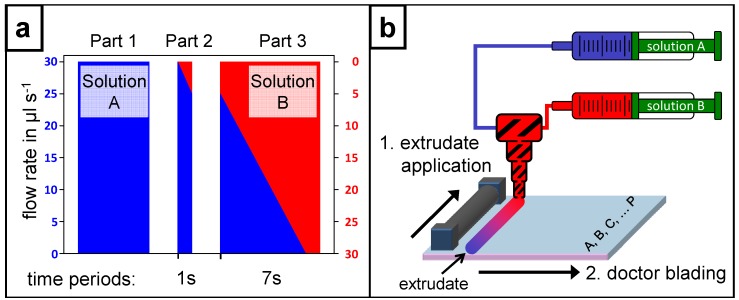
(**a**) Schematic illustration of the flow rate profile controlled by the neMESYS syringe pump system: Part 1 serves as the rinsing step with solution A to get the static mixer air bubble-free; in part 2 the premix inside the static mixer is prepared; part 3 shows the opposite flow rate gradients of the two syringes while the actual extrusion takes place. (**b**) The detailed preparation of a composition gradient film is schematically illustrated. The gradient extrudate of solution A (blue) and solution B (red) (step 1: extrudate application) was realized as described for Figure 1a, part 3. Subsequently the gradient extrudate was doctor bladed perpendicular to the application direction (step 2: doctor blading) and annealed to remove the solvent. For a systematical characterization the yielded film with the internal composition gradient was divided alongside the gradient into the sectors A–P.

For the preparation of a film including an internal composition gradient two individual controllable syringe pumps were used. The syringe pump system allows the preparation of a gradient extrudate made of solutions A and B, when equipped with two syringes. One syringe is filled with solution A and the other with solution B. The syringes are connected via PTFE tubes to the upper part of a static mixer. Both tubes were filled completely with the respective solutions. The static mixer was rinsed just with solution A to ensure the static mixer was air bubble-free (see the flow rate profile in Figure 1a; part 1). Afterwards the syringe pump system was started for one second (see Figure 1a; part 2) to premix both solutions in the fixed volume of the static mixer. This step is necessary to extrude the rinsing solution A from the mixing device and to fill it with the solution of the initial gradient extrudate. Subsequently the static mixer was fixed to the movable part of a doctor blade machine to enable the application of the gradient extrudate on a substrate.

**Figure 2:**
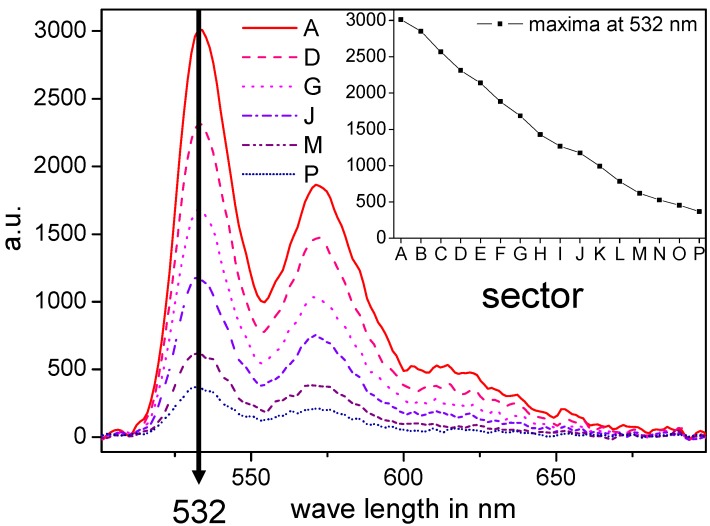
The characterization of the fluorescent dye gradient in PMMA via fluorescence spectroscopy: 16 fluorescence spectra (sector A–P) along the application direction with an equal gap were measured (only six spectra are shown over the full measured wave length range for a better overview). The inset of the graph shows the intensities of the maximum at 532 nm of the spectra of all 16 sectors and verifies distinctly the continuous decrease of the fluorescence intensity given by the material gradient.

The efficiency of this preparation method concerning the constant change of the resulting internal composition gradient film is demonstrated realizing a PMMA film with a gradient of a fluorescence dye. This gradient film was prepared utilizing two 10 wt % solutions of PMMA in THF, while in solution A the fluorescent dye *N,N'*-di(1-heptyloctyl)-perylene-3,4,9,10-bis(dicarboximide) (to exclude concentration quenching: 0.02 mmol in 1 g of solution) was dissolved. The fluorescent dye gradient film was characterized by fluorescence spectroscopy utilizing a fluorescence reader. The acquired fluorescence spectra excited at a wavelength of 455 nm show three maxima at 532 nm, 572 nm and 620 nm (see Figure 2). The observed continuous intensity decrease of the complete spectra demonstrates the prepared internal dye gradient. The intensities of the maximum at 532 nm of all 16 measured spectra of sectors A to P along the composition gradient are summarized in the inset verifying the continuous decrease of the fluorescence dye concentration. In conclusion, this technique offers a simple and efficient way to make a solvent-based gradient extrudate, which withstands doctor blading as well as an ongoing thermal treatment to realize a solid film with an internal composition gradient over a broad concentration range.

#### 2.1.2. Temperature Gradient

Various well defined and long-term stable temperature gradients were engineered in our laboratory on the basis of plates of different metals. On one side of the plate a vessel was welded and filled with, for example, ice-water or liquid nitrogen, which represent the cooling source. Here it has to be considered that the vessel must be refilled continuously to ensure constant cooling. The other side was placed flat on a hot plate, serving as an active heating source, and was adjusted to a desired temperature. After a calibration time of one hour the temperature gradient stayed constant and was verified by an infrared camera. For this a reference silicon wafer used for measuring purposes was placed in the center of the setup’s metal plate. 

**Figure 3:**
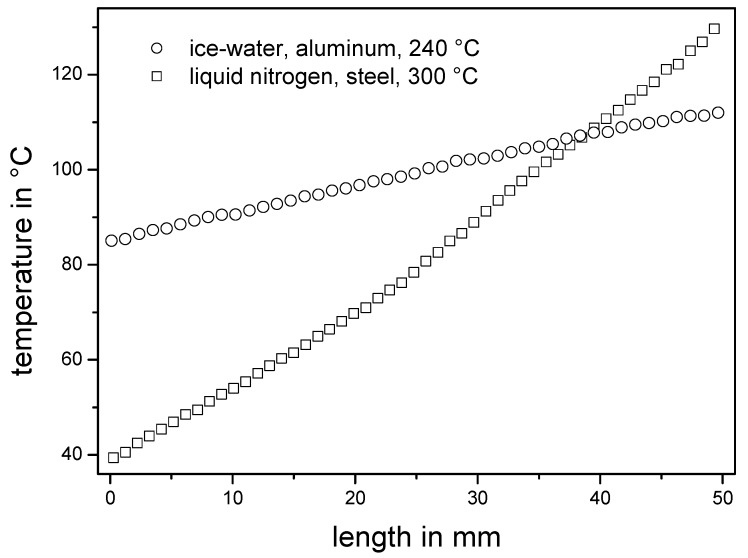
Two temperature profiles—measured using the reference silicon wafer—are shown utilizing different metal plates prepared over the length of 50 mm. The first temperature gradient (circles) was made on an aluminum plate cooling on the left side with ice-water and heating on the right side at the temperature of 240 °C. The achieved temperature gradient shows a steady slope of 0.55 °C/mm from 85 °C to 112 °C. The second (squares) was prepared under the conditions liquid nitrogen, steel plate and 300 °C. It shows an overall slope of 1.85 °C/mm from 39 °C to 130 °C.

In Figure 3 two temperature gradients with different slopes are shown over a length of 50 mm measured using a reference silicon wafer. The gradients demonstrate the ability to adjust the temperature gradients by defining appropriate conditions: for the more flat temperature gradient (circles) ice-water was used for the active cooling, an aluminum plate served as thermoconductive metal plate and for constant heating the hot plate was adjusted to 240 °C. A steeper gradient (squares) was generated with liquid nitrogen as cooling source, a steel plate and the hot plate adjusted to 300 °C.

The flatter temperature gradient (circles) resulted in a steady slope of 0.55 °C/mm from 85 °C to 112 °C. The second temperature gradient (squares) shows a very steep slope of 1.85 °C/mm from 39 °C to 130 °C. Further variations in hot plate temperature, plate material or cooling source allow well-defined temperature slopes in a wide temperature range. The results and customizability demonstrate impressively the applicability of this simple temperature gradient setup and thus offer a versatile utility for annealing processes, e.g., investigations on morphology or thermal crosslinking of polymer films.

#### 2.1.3. Exposure Dose Gradient

The exposure procedure can be the most time-consuming step in manufacturing processes including a light-induced reaction. Combinatorial investigations are an appropriate approach to identify the optimized exposure conditions to save time and to achieve optimal results. Thus combinatorial exposure optimization methods can be used in a versatile manner and easily implemented in exposure investigations of solid films. Exposure dose gradients are conceivable for photocycloaddition, orientation of chromophores and photopolymerization driven by photoexposure and also evaporation-condensation and polymer degradation investigations caused by electron beam exposure.

**Figure 4:**
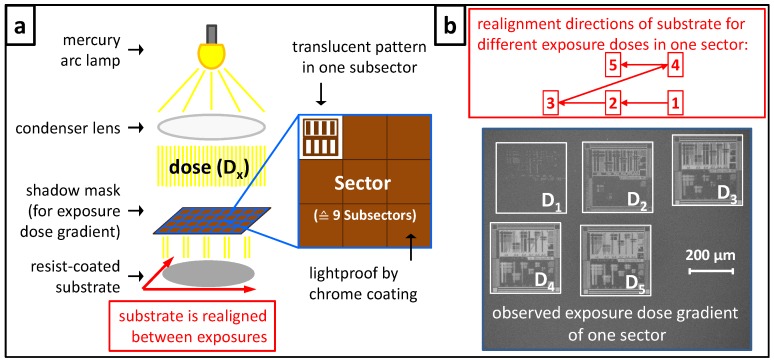
(**a**) The schematic illustration shows a mask-alignment system consisting of the main components: a mercury arc lamp as light source, a condenser lens for the production of parallel light to exposure uniformly a film-coated substrate through the shadow mask. One sector of overall 4,900 sectors is shown in the schematic magnification of the shadow mask, which are arranged and numbered in an X/Y coordinate system. Each sector consists of eight out of nine subsectors with lightproof chrome coating (brown) and one out of nine subsectors of a translucent pattern (white/brown). The substrate holder is adjustable via micrometer adjustment to allow a precise alignment for subsequent exposures. (**b**) Schematic illustration of the realignment directions of the substrate between different exposures: The SEM image shows a selected sector with an exposure dose gradient of five subsectors realized with a positive tone resist (dark grey). The dose was increased from **D_1_** to **D_5_** leading from an under- to an overexposure of the resist shown by the increasing proportion of the bright appearing substrate surface (light grey).

An exposure dose gradient for UV exposure was realized for this work with a mask-alignment system and a specially designed quartz glass mask. Figure 4a shows a schematic illustration of such an exposure set-up with a mercury lamp as light source and a condenser lens to generate parallel light, thus a film coated substrate is uniformly exposed with a dose (**D**) through the shadow mask. This mask consists of 70 × 70 sectors arranged and numbered in an X/Y coordinate system. Each sector has a size of 1.2 mm × 1.2 mm. It is designed with eight out of nine subsectors with a lightproof chrome coating (brown) and one out of nine subsectors of a translucent pattern (white/brown) with an area of 300 µm × 300 µm for each subsector. The mask-alignment system has an alignment stage with micrometer adjustment for the substrate holder. This allows an accurate alignment for subsequent exposures of subsectors in one sector on the same substrate. Therefore the substrate is moved by 400 µm for realignment between two exposures obtaining two subsectors; by this the substrate is moved 100 µm further than the edge length of the translucent pattern to avoid areas of inadvertent double exposure. For the verification of the UV-exposure gradient a positive tone resist was used. The procedure for a dose-gradient exposure was conducted as follows: the film-coated substrate was adjusted to the substrate holder in the mask-alignment system and the exposure of the first subsectors in all sectors was conducted. Subsequently the substrate is realigned by movement of the substrate holder of 400 µm to the left defining the position for the second subsectors’ exposure and again 400 µm to the left for the third exposure (see Figure 4b).

For the fourth exposure the substrate holder is realigned to the origin (800 µm to the right) and then 400 µm up and for the fifth exposure 400 µm to the left. The dose was increased for every exposure step from 80 mJ/cm² to 160 mJ/cm² and as a result a dose gradient of five different doses on an area of 1.44 mm² was created. Subsequently the film-coated substrate was thermally treated at 105 °C for 30 s for post exposure bake. Afterwards the film is developed in an aqueous 0.26 N tetramethylammonium hydroxide (TMAH) solution for 30 s resulting in the removal of the exposed resist if the dose was sufficiently high. The exposure dose gradient of a selected sector is shown in the SEM image in Figure 4b: pattern (**D_1_**) exposed with the lowest dose is obviously underexposed as the contours of the pattern are just slightly visible and the resist (dark grey) is roughly developed. Increasing the dose results in more clearly developed patterns, indicated by the stronger contrast (dark–bright) in the corresponding subsectors given by the undeveloped unexposed resist material beside the substrate surface (light grey). At the highest dose (**D_5_**) the pattern is overexposed, which is obvious by the fact the features are beginning to strip off.

**Figure 5:**
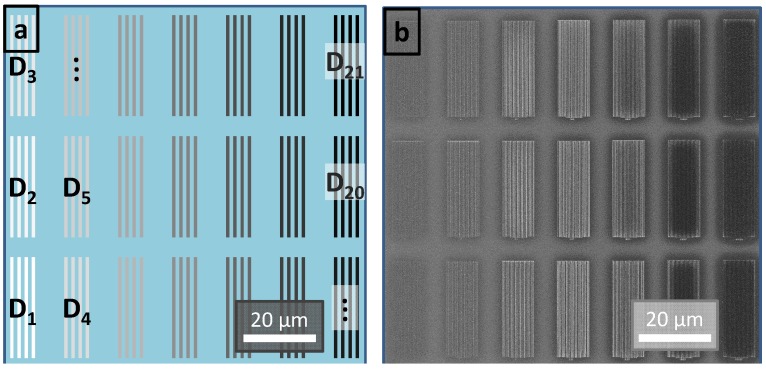
(**a**) The schematic illustration shows a write-field with an area of 100 µm × 100 µm used for electron beam lithography. The write-field consists of 21 arrays of a 100 nm line/space pattern with an increasing dose from bottom left (**D_1_**) to the upper right (**D_21_**) indicated by the greyscale gradient. (**b**) The SEM image shows the dose gradient of line/space patterns of a developed negative tone resist of a complete write-field. **D_1_** is underexposed and just shadowy observable on the substrate. The patterns get more visible because of the increasing dose: in the center the pattern are exposed to their optimum dose while at higher doses the patterns get overexposed and even besides the pattern some resist material remains, observable by the darker arrays at these high doses.

For electron beam exposure a different approach is necessary for realizing an exposure dose gradient. The coated resist film is not exposed through a mask in one exposure step, but rather it is continuously exposed by a controllable electron beam. For this so-called direct write technique the patterns are arranged in write-fields. In Figure 5a shows an example of a write-field of 100 µm × 100 µm in size, which contains 21 arrays of a 100 nm line/space pattern. Each array is written by a defined programmable exposure dose. When beginning with the lowest dose for the first array (**D_1_**) and continuing with increasing doses realized by the multiplication with a selected constant, an exposure dose gradient is generated.

In Figure 5b the SEM image shows a selected section of the resulting exposure dose gradient of a film prepared out of a negative tone resist: The pattern corresponding to the lowest dose (**D_1_**) in the bottom left is only shadowy observable and is clearly underexposed. Increasing the dose results in more distinct patterns and the optimum dose range is observed in the center. A further dose increase results in overexposed patterns (right), indicated by residues surrounding the actual patterns and observable by the darker arrays.

Exposure dose gradients for deep UV and electron beam exposure realized by the combinatorial approaches described above are not limited to the lithographic investigations shown but are also suitable for process optimization of exposure processes.

#### 2.1.4. Dissolution Investigation

The dissolution behavior of organic film systems in distinct organic solvents or aqueous media is of fundamental and essential interest for many scientific investigations and technical applications. Thus we used quartz crystal microbalance (QCM) measurements as a screening method to investigate the impact of solvents or solutions to time-resolved swelling and/or dissolution behavior of organic thin films. Here we demonstrate the efficiency of this method with respect to the dissolution behavior of a polymer resist film by increasing stepwise the base strength of an aqueous tetramethylammonium hydroxide (TMAH) solution. 

**Figure 6:**
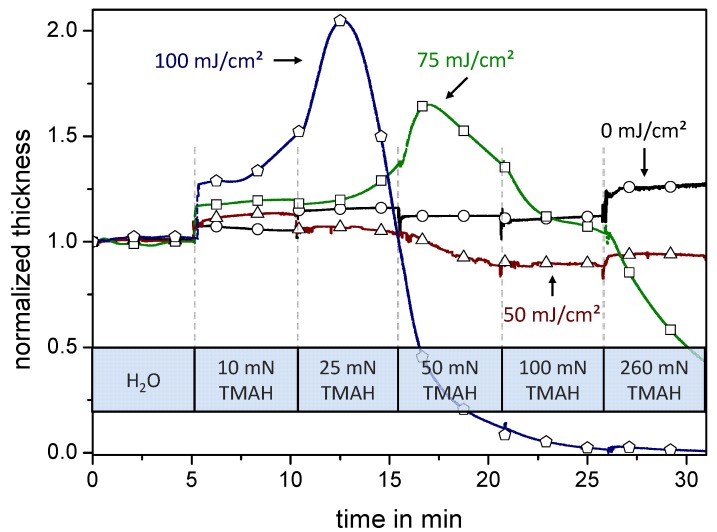
Quartz crystal microbalance (QCM) measurements of four resist films exposed to different UV exposure doses are shown. The graphs show the corresponding QCM frequencies change—translated to normalized film thickness change [26]—during the periodic titration of a concentrated developer solution observable due to the transient oscillation. The unexposed hydrophobic resist film (○) stays at a nearly constant film thickness while slight swelling and dissolving of reacted resist material is observable for the resist film exposed to 50 mJ/cm² (△). The resist film exposed to 75 mJ/cm² (□) shows a more distinct swelling character. The swelling slightly begins at the first titration and increases markedly until the concentration of 50 mN where the dissolving character exceeds. This behavior of swelling and dissolving is also observed for the resist exposed to 100 mJ/cm² (⌂) but the dissolution shifts to a lower concentration of 25 mN. The exposed resist film is fully developed at a concentration of 100 mN TMAH.

For this purpose films on quartz crystals (QCs) were prepared out of a positive tone resist and a photoacid generator (PAG). Polymer-coated QCs were exposed to UV-light to activate the PAG, followed by a PEB step to catalyze the deprotection reaction. Thus, depending on exposure time and consequently the amount of activated PAG, the resist becomes hydrophilic. These selectively exposed QCs were clamped one by one into the QC holder and immersed into water during dissolution measurement. After defined time periods a stepwise increase of the TMAH concentration was achieved from 10 to 25, 50, 100, and 260 mN by adding corresponding amounts of a concentrated TMAH solution (see Figure 6).

Figure 6 shows film thickness changes of resist films exposed to different UV doses during their development. In addition, the stepwise increase of developer strength after defined time periods allows detailed investigations on dissolution behavior with relation to dissolution time and the developer (TMAH) concentration at a fixed exposure dose. All resist films show no swelling or dissolving characteristics in pure water at the beginning of the measurement. At the first titration a sudden increase of film thickness for all resist films is observable, explainable by the deprotonation of the acrylic acid units. Here, the carboxylate anions formed result in repulsion and in an ambient hydration which dampens the oscillation frequency of the quartz crystal. In addition, this measurement method is so sensitive that the distinct density increase is detected by a general oscillation frequency damp at the last titration step from 100 mN to 260 mN. The unexposed film (0 mJ/cm²) retains a constant film thickness over the complete titration process. Here all titration steps can be easily identified by the frequency jumps. However the lowest exposed resist film (50 mJ/cm²) shows a slight swelling character at the first titration step as well as a slow dissolution occurs at a concentration of 50 mN. The resist film exposed to 75 mJ/cm² shows a more distinct swelling character at the first titration step and shows a swelling maximum at the TMAH concentration of 50 mN followed by the dissolution of the resist material observable by the increasing oscillation frequency. The most exposed resist film (100 mJ/cm²) shows a high swelling character already at the first titration step and reaches the maximum at a concentration of 25 mN TMAH. The following fast dissolution of the resist film ends in a fully developed quartz crystal at the concentration of 100 mN TMAH.

The results of the QCM prescreening experiments specify the processing window of the dissolution behavior of investigated organic materials. Typically the materials’ dissolution is influenced by the nature of the solvent as well as the application period. In this context a simple way to study dissolution behavior is an immersion process, which allows also the preparation of gradients controlled by the immersion period. Two options are conceivable to realize an immersion time gradient: stepwise or continuous: for the stepwise immersion time gradient it is useful to section the substrate into a defined number of segments via marks at the edge of the substrate. The substrate is clamped into an inverse pair of tweezers adjusted above a filled vessel and immersed then into the solution beginning at the lowest segment. In consequence the lowest segment gets the longest immersion time period in contrast to the top segment which gets the shortest one. After the solution treatment the substrate is removed from the vessel all at once. Dependent on the application of this immersion gradient the film on the substrate must be dried, immersed into another solution or the film must be rinsed with an inert solvent.

A continuous immersion gradient was realized with an electrical motor drive which has ten adjustable speeds from 25 µm/s to 25 mm/s. A wire was attached to the drive roller of the motor drive and connected with the further end to an inverse tweezers which again clamps the substrate. The drive roller was positioned directly above the filled vessel the attached substrate has to be immersed into. Then the substrate was lowered into the vessel at a defined speed. The time intervals of the solution treatment for each position on the substrate are calculated by the length of the immersed substrate and the preset speed of the electrical motor drive. After complete immersion, the substrate was removed from the solution all at once and treated as described above.

### 2.2. Combinatorial Libraries

Synthesis and/or investigation of applicability of new materials are often time-consuming and take a lot of effort. Thus, it is a common approach to use combinatorial techniques as a fast way for getting materials and optimizing their processing variables. However, the full potential of a new material—be it the synthesis or the properties—is typically affected by the interaction of multiple variables, so the possibility of material optimization of just one variable will result only in a local optimum. To avoid this fact, a combinatorial investigation of at least two interacting variables should be conducted in one investigation, a so-called combinatorial library.

#### 2.2.1. Binary Combinatorial Library

In the following we show an example for the combination of two gradients in one combinatorial library. Therefore a film with an internal composition gradient is applied on a substrate and annealed with a temperature gradient perpendicular to it. These two variables are crucial for lithographic systems, especially as process variables for chemically amplified resists. For the experiment presented here a resist system known from the literature [27] was used, consisting of α,α,α'-tris(4-hydroxy-phenyl)-1-ethyl-4-isopropylbenzene (as matrix component), N,N,N,N-tetra-(methoxymethyl)glycoluril (as crosslinker component), and triphenylsulfonium perfluoro-1-butanesulfonate (as photoacid generator—PAG). The efficiency of a well-designed combinatorial library is next shown using this known three component resist system.

**Figure 7:**
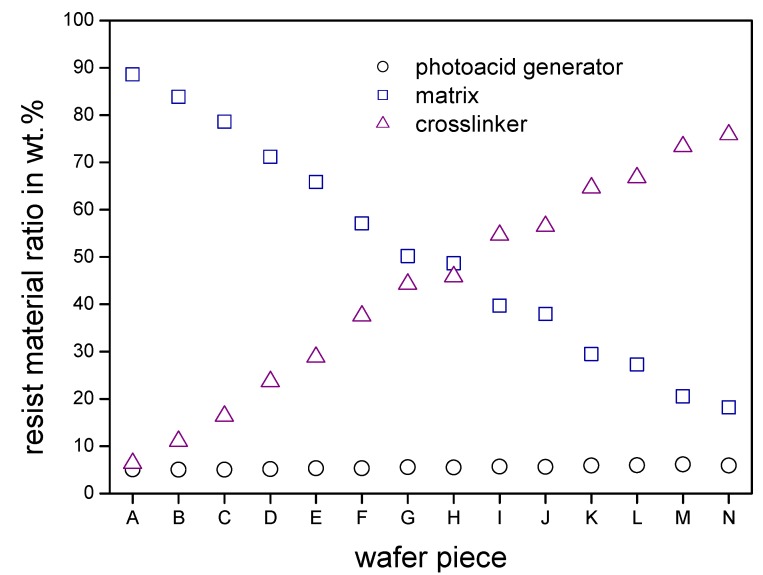
The graph shows the resist material ratios of the 14 wafer pieces of the three components resist gradient film analyzed by high performance liquid chromatography. In the achieved material composition gradient the photo acid generator content (○) stays constant in the desired range of five to six weight percent. The matrix content (□) is constantly decreasing from 88.6 wt % to 18.2 wt % while the crosslinker content (△) is steadily increasing from 6.4 wt % to 75.9 wt %.

For the film preparation a silicon wafer of four inch diameter served as substrate. The resist film consisted of an internal composition gradient of matrix component *versus* crosslinker component with an overall constant concentration of PAG, as well as an overall constant film thickness. This film was applied out of solution A (9.5 wt % of the matrix component + 0.5 wt % of PAG in 1-methoxy-2-acetoxypropane) and solution B (9.5 wt % of the crosslinker component + 0.5 wt % of PAG in 1-methoxy-2-acetoxypropane). The resist material gradient was prepared as described in Section 2.2.1, followed by a subsequent thermal prebake step at 115 °C for 60 s. For analyzing the material composition of the resulting gradient, high performance liquid chromatography (HPLC) was used. Beforehand the three components were calibrated to determine weight ratios of component mixtures. From the prepared composition gradient on the silicon substrate a defined stripe was cut off with a length of 70 mm and a width of 10 mm parallel to the application direction. The stripe was divided into 14 pieces (**A**–**N**) with a length of 5 mm and each was rinsed off with acetonitrile separately for the HPLC analysis. The measured ratios of the three components are shown graphically in Figure 7 and detailed values listed in Table 1.

The composition gradient produced by gradient extrusion and subsequent doctor blading was realized over a broad concentration range with a nearly constant film thickness of 350 nm: the PAG concentration stays nearly constant at 5–6 wt % over the whole length of 70 mm. The matrix content is continuously decreasing from 88.6 wt % to 18.2 wt % while the crosslinker content is steadily increasing from 6.4 wt % to 75.9 wt %. The gradient covers a huge range of resist component ratios and thus presents a good starting point for a combinatorial investigation of a multicomponent material system.

**Table 1:** Material composition ratios of each wafer piece of the three components resist gradient film analyzed by high performance liquid chromatography.

Wafer piece	A	B	C	D	E	F	G	H	I	J	K	L	M	N
Wafer segment (mm)	0–5	5–10	10–15	15–20	20–25	25–30	30–35	35–40	40–45	45–50	50–55	55–60	60–65	65–70
PAG (wt %)	5.0	5.0	5.1	5.1	5.3	5.3	5.5	5.5	5.7	5.6	5.9	5.9	6.2	5.9
Matrix (wt %)	88.6	83.9	78.6	71.2	65.9	57.1	50.2	48.7	39.7	37.9	29.5	27.3	20.5	18.2
Crosslinker (wt %)	6.4	11.1	16.3	23.7	28.8	37.6	44.3	45.8	54.6	56.5	64.6	66.8	73.3	75.9

**Figure 8:**
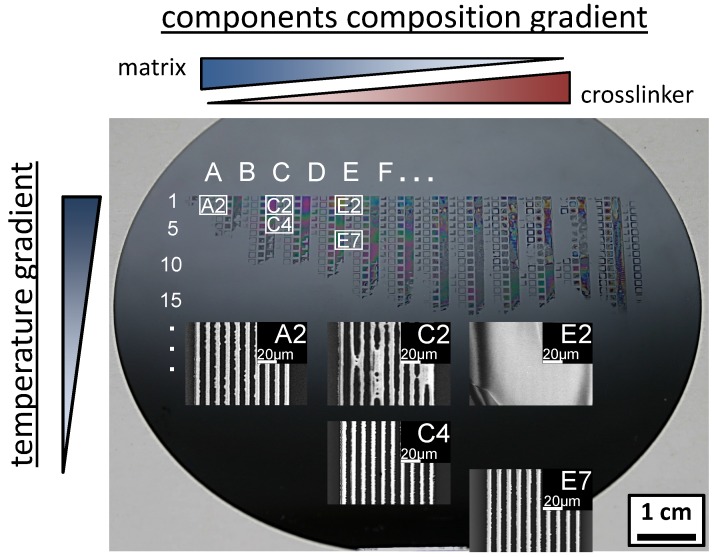
Photograph of the patterned resist film on the silicon wafer after applying the material composition gradient (left to right: column **A** to **N**), post apply bake of 115 °C for 60 s, photo exposure (60 mJ/cm²), PEB temperature gradient (top to bottom: row **1** to **40**) and development in a 0.26 N aqueous solution of tetramethylammonium hydroxide for 10 s. Already with the naked eye can be seen that below row **15** (60 °C) all patterns are completely stripped off. The dependence of temperature to material composition is obvious by the SEM insets of column **E**: sector **E7** shows distinct pattern while in sector **E2** all lines are merged. Under the conditions of a steady temperature (row **2**) distinct pattern are obvious at high matrix content (sector **A2**) while with increasing crosslinker content the patterns tend to merge (**C2**/**E2**). A trend is observable as for higher crosslinker content lower PEB temperatures are needed for distinct patterns (**A2**, **C4**, **E7**).

The next step in the lithographic process is the exposure, which activates the PAG. This step was conducted with the EVG^®^620 mask-aligner utilizing a shadow mask with a line/space pattern. The dose was adjusted to 60 mJ/cm². Immediately afterwards a PEB was applied to perform the acid catalyzed crosslinking reaction of crosslinker and matrix. For the preparation of a combinatorial library a temperature gradient for PEB was applied perpendicular to the material composition gradient for 30 s. The applied temperature gradient had a constant slope of 1.07 °C/mm within the temperature interval from 85 °C to 20 °C. Subsequently the resist film was developed in a 0.26 N aqueous solution of TMAH for 10 s by dissolving the resist materials of the unexposed areas. In Figure 8 a photograph of the processed film with the remaining patterned resist material and SEM images of the pattern of selected sectors of the combinatorial library are shown.

The composition gradient ranges from a high matrix content (left: column A) to a high crosslinker content (right: column N) arranged perpendicular to the temperature gradient from a high (top: row 1) to a low temperature (bottom: row **40**). It can be seen with the naked eye that below row **15** (60 °C) even the exposed resist of each material composition was completely stripped off the substrate during development. This indicates a minimum temperature is necessary to crosslink this resist system at any material composition. Most of patterns to the right of column **F** (matrix ≤ 57.1 wt %/crosslinker ≥ 37.6 wt %/PAG 5 wt %) corresponding to high crosslinker contents are stripped off, too. The inset SEM images show clearly the PEB temperature dependence on the resist material composition (column **E**): While sector **E7** (72 °C) shows distinct lines, in sector **E2** (83 °C) the pattern lines are merged together. This is due to the fact that the higher applied temperature results in an increased acid diffusion to this material composition beyond the exposed areas. With a higher matrix component (row **2**) the pattern quality increases from sector **E2** to **C2** until in sector **A2** distinct patterns were observed. These observations reveal a tendency which is confirmed by sector **C4**: distinct patterns were achieved with decreasing matrix content and concurrent temperature decreases beginning with sector **A2** over sector **C4** and ending with sector **E7**. These results demonstrate impressively the strength of combinatorial libraries in recognizing variable dependent trends of interacting multi-variable system and so combinatorial approaches are an excellent technique for optimizing material composition and their processing conditions.

#### 2.2.2. Ternary Combinatorial Library

The upgrade of a binary combinatorial library by a third gradient is here called ternary combinatorial library. For the preparation of a binary combinatorial library variable gradients are typically arranged orthogonally on two-dimensional areas. Thus for the realization of a ternary library the third variable gradient must be implemented in the third dimension or arranged in a very small area in which the first as well as the second variable are effectively constant within the gradient. To implement the latter case the third variable is applied several times—matrix-like—in one small spot on the binary library. As described above the exposure dose gradients have the ability to fulfill these requirements and hence it is possible to investigate three interacting variables in just one combinatorial library.

**Figure 9:**
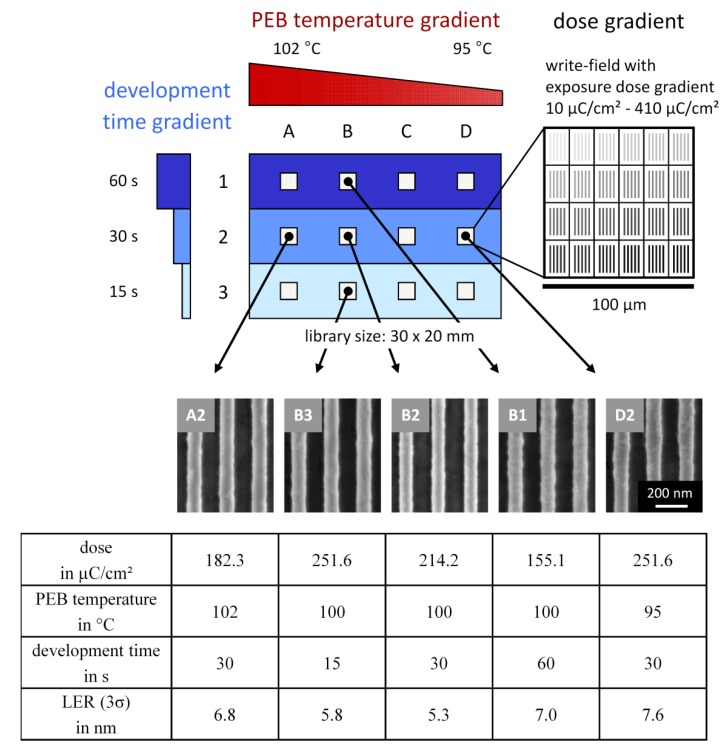
Schematic illustration of the ternary combinatorial library prepared on a substrate with a size of 30 mm × 20 mm. The PEB temperature gradient (95–102 °C) is arranged horizontal and perpendicular to it the development time step gradient (15 s, 30 s, 60 s). The 12 write-fields (100 µm × 100 µm) with the exposure dose gradient (10–410 µC/cm²) are arranged matrix-like in small sectors and provide 24 arrays of 100 nm line/space profiles. The selected SEM images demonstrate the strong influence of the applied three variable gradients. The respective conditions (PEB temperature, development time, and dose plus the LER values) of these patterns are listed below.

In the following we present the developed ternary library for a lithographic investigation as shown schematically in Figure 9. The ternary library consists of a temperature gradient perpendicular to a development time gradient, which represents the variable gradients of the binary library, while small sectors of electron beam exposure dose gradients are applied matrix-like to this binary combinatorial library. For the shown lithographic combinatorial investigation a star-shaped teroligomer [statistically copolymerized out of γ-butyrolactone methacrylate, methyladamantyl methacrylate, and hydroxyladamantyl methacrylate (GBLMA-co-MAMA-co-HAMA)] as positive tone resist plus the PAG triphenylsulfonium perfluoro-1-butanesulfonate was used. The resist material solution was cast via spin coating on a hexamethyldisilazane-primed four inch silicon wafer, resulting in a film thickness of about 90 nm. The film-coated substrate was cut to a rectangle of 30 mm × 20 mm, thermally treated at 125 °C for 150 s, and electron beam exposed by a dose gradient which was conducted in 12 write-fields, applied matrix-like (see Figure 9). This matrix consists of three rows and four columns while the gap between each row is 10 mm and between each column 5 mm. Each write-field—100 µm × 100 µm in size—comprises an exposure dose gradient of 24 arrays of 100 nm line/space pattern between 10 µC/cm² to 410 µC/cm². Afterwards PEB was applied—parallel to the rows of the exposed matrix—by a temperature gradient for 30 s. This temperature gradient was prepared on an aluminum plate with ice-water as cooling source and a hot plate adjusted to 220 °C as heating source. Thus the temperature gradient applied to the substrate ranged from 95 °C to 102 °C with a slope of 0.47 °C/mm. After that a development time step gradient was applied alongside the columns of the exposed matrix for 15, 30, 60 s in a 0.26 N tetramethylammonium hydroxide solution. Therefore the substrate was subdivided into three segments whereas each segment contains one row of write-fields. Selected SEM images of line/space patterns and their corresponding processing conditions are shown in Figure 9. Furthermore line edge roughness (LER) values – used as performance criterion—of these selected line/space patterns are also listed. LER is a mathematical calculated value, describing the deviation of pattern edges to an ideal shaped pattern. The LER values of the line/space patterns were calculated over a length of 15 µm by the software SuMMIT^TM^ from EUV technology.

The extensive scanning electron microscopy investigation of the ternary combinatorial library showed clear dependencies on the applied variables exposure dose, PEB temperature, and development time. The distinct quality of the observed line patterns guided us straight forward to the optimized sectors and the LER values identify the detailed influences of these variables in dependence to the resist performance. The pattern with the clearest lines (**B2**) was observed at a dose of 214.2 µC/cm², a PEB temperature of 100 °C, and a development time of 30 s resulting in a LER value of 5.3 nm. This achieved LER of the investigated statistical star block copolymer is clearly decreased compared to a former published reference linear terpolymer with a LER value of about 7 nm [25] and thus demonstrates the efficiency of this combinatorial approach to identify the potential of a new resist material within one ternary combinatorial library. The patterns prepared at a PEB temperature higher and lower than the observed optimum temperature of 100 °C, but with the same development time show clearly increased LER values of 6.8 nm (**A2**) and 7.6 nm (**D2**). Noticeable is the decreasing dose required to achieve pattern for a higher applied PEB temperature (**A2**) under the same development conditions. Less PAG was activated due to a lower dose, but this is compensated by the increased acid diffusion and reaction rate at higher temperatures and vice versa for **D2**. The increase of LER values are also observable for patterns annealed at the same PEB temperature but applied to longer and shorter development times than the observed optimum of 30 s with the LER of 5.3 nm in sector **B2** (**B1**: 60 s, LER 7.0 nm, **B3**: 15 s, LER 5.8 nm). Here the operation with the optimized exposure dose is distinctive and crucial. While the exposure dose for the longest development time **B1** is about 25% lower than the dose of the optimum **B2**, the dose for the shortest development time **B3** is about 20% higher than the dose of the optimum pattern in **B2**. In summary, this combinatorial investigation demonstrates the strong interdependence and interaction of resist processing variables and shows the high efficiency of a ternary combinatorial library for such a complex investigation of a multi-variable system.

## 3. Experimental

### 3.1. Chemicals and Materials

Unless otherwise stated, all solvents and chemicals were purchased from Sigma-Aldrich (St. Louis, MO, USA) and used as received. *N,N'*-Di(1-heptyloctyl)-perylene-3,4,9,10-bis(dicarboximide) was kindly provided by Dr. Andrè Wicklein, MC I, University of Bayreuth. Poly (methyl methacrylate) (M_w_ = 123.4 kg/mol) was purchased from Evonik (formerly known as Degussa, Essen, Germany). 1,1,1-tris(4-hydroxy­phenyl)-1-ethyl-4-isopropylbenzene was purchased from ABCR (Karlsruhe, Germany). *N,N',N'',N'''*-tetra(methoxymethyl)glycoluril was purchased from Worlée-Chemie (Hamburg, Germany).

### 3.2. Internal Material Composition Gradient

The gradient extrudate was prepared using the neMESYS syringe pump system (cetoni GmbH, Korbußen, Germany) containing two individual controllable syringe pumps which were connected via PTFE tubes to a static mixer (Adchem GmbH, Wendelstein, Germany; MA 3.0-13-S: shortened to a length of 15 mm). The syringe pump system was programmed to start solution A with a flow rate of 30 µL/s but decreasing constantly with time, while solution B starts with a flow rate of 0 µL/s but increasing constantly with time. For the actual extrusion of the homogeneous mixed gradient the flow rates of each syringe pump are tuned regarding a summarized steady flow rate of 30 µL/s, thus a constant variation of the solutions A & B is given for a time period of seven seconds (see Figure 1a; part 3). Simultaneously the doctor blade machine to which the static mixer is fixed has to be activated with a velocity of 10 mm/s for the same time period. Both simultaneously running procedures are described in Figure 1b step 1: extrudate application. The second step takes place after the substrate’s rotation through 90 degrees. The extrudate is then doctor bladed with a doctor blade machine (Erichsen Coatmaster 509 MC-1, Hemer, Germany) using a 4-sided bar (BYK, Wesel, Germany; gap: 50, 100, 150, 200 µm) perpendicular to its application direction with a velocity of 10 mm/s using a doctor blade of corresponding size (see Figure 1b step 2: doctor blading). Afterwards the film was baked to evaporate the application solvent and to fix the composition gradient. The yielded film with the internal composition gradient was divided alongside the gradient into the sectors A to P for a systematical characterization. The PMMA film with the internal gradient of the fluorescent dye was verified by Fluorescence Reader Flashscan 530 (AnalytikJena AG, Jena, Germany). The measurement was executed at spots every 5 mm parallel to the gradient. The resist film with the internal composition gradient was verified with high performance liquid chromatography (HPLC; Agilent 1100 series, Böblingen, Germany, column: ZORBAX Bonus-RP 4.6 × 150 mm, 5 µm; 1 mL/min of 80% ACN/20% H2O; 201 nm/278 nm).

### 3.3. Temperature Gradient

The temperature gradients were adjusted on metal plates with a length of 35 cm, a width of 20 cm, and a thickness of 0.5 cm. On one side of the plate a vessel was welded with a length of 10 cm, a width of 20 cm, and a height of 10 cm serving as reservoir of the cooling agent. This cooled side of the metal plate was placed on cork for insulation. The other side of the plate was placed planar on a hot plate (Präzitherm PZ 28-2T; Harry Gestigkeit GmbH, Düsseldorf, Germany) with a defined contact area of 6 cm × 20 cm. A reference silicon wafer—525 µm thickness, polished front side, n-type, highly arsenic doped with a resistance of <0.01 Ωcm, which exhibits less infrared transparency—was placed on the plate after a calibration time of one hour. This wafer was monitored with an infrared camera (ThermaCAM^TM^E300; FLIR Systems, Wilsonville, OR, USA), which was adjusted 30 cm directly above the wafer. Before utilizing the infrared camera, the extinction coefficient of the silicon wafer was calibrated with the help of the melting points of stearic acid (69 °C), benzyl (1,2-diphenylethane-1,2-dione) (95 °C) and benzoic acid (122 °C) [28].

### 3.4. Exposure Dose Gradient

The UV-exposure dose gradient using a mask-alignment system (EVG^®^620; EV Group, St. Florian am Inn, Austria) and specially designed five inch quartz glass mask (produced by ML&C, Jena, Germany). For the verification of this UV-exposure gradient a resist film was prepared out of a positive tone star-shaped teroligomer [statistically copolymerized out of γ-butyrolactone methacrylate, methyl adamantyl methacrylate, and hydroxyl adamantyl methacrylate (GBLMA-co-MAMA-co-HAMA)] (95 wt %) and the photoacid generator triphenylsulfonium perfluoro-1-butanesulfonate (5 wt %) and was applied from a 2.5 wt % solution in 1-methoxy-2-acetoxypropane on a silicon wafer. After spin coating the resulting film was pre-baked and had a thickness of 90 nm measured by a surface profilometer (Dektak 3030 ST; Veeco, Plainview, NY, USA). The wavelength range of the mask-alignment system was adapted to 240 nm to 290 nm for the used resist system. The electron beam exposure was performed using a Zeiss 1530 FESEM equipped with a Raith Elphy Plus at an accelerating voltage of 20 kV and doses were adjusted from 100 µC/cm² (**D_1_**) to 2516 µC/cm² (**D_21_**). The exposure took place on a new molecular glass negative tone resist utilizing physical vapor deposition for film preparation and consisted of 2-[(methylsulfonyl)oxy]-1*H*-benz[de]isoquinoline-1,3(2*H*)-dione (41%), 1,1'-binaphthyl-2,2'-diamine (40%), and 2-[(methyl­sulfonyl)oxy]-1*H*-benz[f]isoindole-1,3(2*H*)-dione (19%). Afterwards the film was developed 60 s in stirred cyclohexane.

### 3.5. Dissolution Investigation

For QCM measurements quartz crystal holder (Maxtek CHC-100, INFICON, Bad Ragaz, Switzerland) and quartz crystals (QCs; 1 inch, 5 MHz, polished gold electrodes; purchased from QT Quarztechnik GmbH, Daun, Germany) were used. For the resist film preparation on the QCs solutions out of a positive tone star-shaped teroligomer (GBLMA-co-MAMA-co-HAMA) and the photoacid generator (triphenylsulfonium perfluoro-1-butanesulfonate) were spin-coated under equal conditions (see Section 3.4) on quartz crystals resulting in a film thickness of about 90 nm. The wavelength for UV-exposure was adjusted to 240 nm to 290 nm (F300S; Fusion UV, Gaithersburg, MD, USA). The PEB was performed at a temperature of 130 °C for 30 s.

## 4. Conclusions

This work demonstrates several methods for combinatorial investigations on solution-coated organic thin films. In this context film preparation of an internal material composition gradient by a syringe pump system and a static mixing device was successful established for polymeric as well as low molecular weight materials. The characterization by fluorescence spectroscopy as well as HPLC confirmed a linear slope of the achieved material composition gradient. We have also shown the controlled preparation of various temperature gradients, here applied for thermal annealing processes whose slope is adjustable to the particular requirement. In addition, we have applied exposure dose gradients for UV light and electron beam exposure on tiny areas allowing a matrix-like application and thus the preparation of a ternary combinatorial library. For dissolution investigations quartz crystal microbalance measurements in combination with a titration process were shown to provide an efficient screening technique. Based on these valuable results further combinatorial investigations on solution-coated films were conducted with either stepwise or continuous immersion gradients. Finally these gradients were combined to a binary and a ternary combinatorial library and applied to lithographic systems. Here the resist material was efficiently prescreened allowing the evaluation of the interdependence of the applied variables. Thus, by this method a fast optimization of the resist material processing was also achieved. These results impressively demonstrate the efficiency of combinatorial approaches on organic thin films applied for complex multi-variable dependent applications.
